# Associations of longitudinal height and weight with clinical outcomes in pediatric kidney replacement therapy: results from the ESPN/ERA Registry

**DOI:** 10.1007/s00467-023-05973-3

**Published:** 2023-05-08

**Authors:** Marjolein Bonthuis, Sevcan A. Bakkaloglu, Enrico Vidal, Sergey Baiko, Fiona Braddon, Carmela Errichiello, Telma Francisco, Dieter Haffner, Annie Lahoche, Beata Leszczyńska, Jurate Masalkiene, Jelena Stojanovic, Maria S. Molchanova, George Reusz, Adela Rodriguez Barba, Alejandra Rosales, Sanja Tegeltija, Elisa Ylinen, Galia Zlatanova, Jérôme Harambat, Kitty J. Jager

**Affiliations:** 1 ESPN/ERA Registry, Department of Medical Informatics, Amsterdam UMC location University of Amsterdam, Meibergdreef 9, Amsterdam, the Netherlands; 2 Amsterdam Public Health, Quality of Care, Amsterdam, the Netherlands; 3 Pediatric Nephrology, Gazi University Faculty of Medicine, Ankara, Turkey; 4 Pediatric Nephrology, Dialysis and Transplantation Unit, Department of Woman’s and Child’s Health, University Hospital of Padua, Padua, Italy; 5 Department of Pediatrics, Belarusian State Medical University, Minsk, Belarus; 6 UK Renal Registry, Bristol, UK; 7 Nephrology and Dialysis Unit, Meyer Children’s Hospital, Florence, Italy; 8 Department of Pediatric Nephrology, Centro Hospitalar Universitário de Lisboa Central, Lisbon, Portugal; 9 Department of Pediatric Kidney, Liver and Metabolic Diseases, Hannover Medical School, Hannover, Germany; 10 Department of Pediatric Nephrology, CHRU de Lille, Lille, France; 11 Department of Pediatrics and Nephrology, Medical University of Warsaw, Warsaw, Poland; 12 Department of Children Diseases, Medical Academy, Lithuanian University of Health Sciences, Kaunas, Lithuania; 13 Department of Paediatric Nephrology, Great Ormond Street Hospital for Children, NHS Foundation Trust, London, UK; 14 Pirogov Russian National Research Medical University, Moscow, Russia; 15 1st Department of Pediatrics, Semmelweis University Budapest, Budapest, Hungary; 16 Pediatric Nephrology Unit, Hospital Universitario Virgen del Rocío, Sevilla, Spain; 17 Department of Pediatrics I, Medical University of Innsbruck, Innsbruck, Austria; 18 Department of Pediatric Nephrology, University Children’s Hospital, Belgrade, Serbia; 19 Department of Pediatric Nephrology and Transplantation, New Children’s Hospital, University of Helsinki and Helsinki University Hospital, Helsinki, Finland; 20 Department of Pediatric Nephrology, University Children’s Hospital “Prof. Ivan Mitev”, Sofia, Bulgaria; 21 Pediatric Nephrology Unit, Bordeaux University Hospital, Bordeaux, France

**Keywords:** Growth, Body composition, Mortality, Kidney transplantation, Children

## Abstract

**Background:**

Associations between anthropometric measures and patient outcomes in children are inconsistent and mainly based on data at kidney replacement therapy (KRT) initiation. We studied associations of height and body mass index (BMI) with access to kidney transplantation, graft failure, and death during childhood KRT.

**Methods:**

We included patients < 20 years starting KRT in 33 European countries from 1995–2019 with height and weight data recorded to the ESPN/ERA Registry. We defined short stature as height standard deviation scores (SDS) < –1.88 and tall stature as height SDS > 1.88. Underweight, overweight and obesity were calculated using age and sex-specific BMI for height-age criteria. Associations with outcomes were assessed using multivariable Cox models with time-dependent covariates.

**Results:**

We included 11,873 patients. Likelihood of transplantation was lower for short (aHR: 0.82, 95% CI: 0.78–0.86), tall (aHR: 0.65, 95% CI: 0.56–0.75), and underweight patients (aHR: 0.79, 95%CI: 0.71–0.87). Compared with normal height, patients with short and tall statures showed higher graft failure risk. All-cause mortality risk was higher in short (aHR: 2.30, 95% CI: 1.92–2.74), but not in tall stature. Underweight (aHR: 1.76, 95% CI: 1.38–2.23) and obese (aHR: 1.49, 95% CI: 1.11–1.99) patients showed higher all-cause mortality risk than normal weight subjects.

**Conclusions:**

Short and tall stature and being underweight were associated with a lower likelihood of receiving a kidney allograft. Mortality risk was higher among pediatric KRT patients with a short stature or those being underweight or obese. Our results highlight the need for careful nutritional management and multidisciplinary approach for these patients.

**Graphical abstract:**

**Figure:**
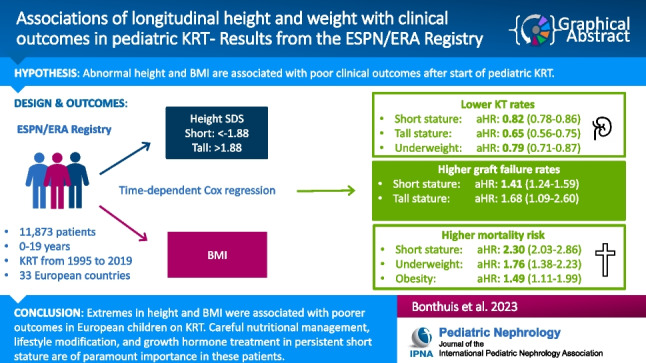
A higher resolution version of the Graphical abstract is available as Supplementary information

**Supplementary Information:**

The online version contains supplementary material available at 10.1007/s00467-023-05973-3.

## Introduction

Mortality risk in pediatric patients on kidney replacement therapy (KRT) remains at least 10 times higher compared with the general pediatric population [1]. Patient survival in pediatric KRT is multifactorial [2, 3] and anthropometric measures such as height and body mass index (BMI) are also associated with increased mortality [4–6]. Furthermore, abnormal height and body composition in pediatric KRT have been associated with other poor clinical outcomes, such as lower quality of life [7], lower access to kidney transplantation and lower graft survival [4–6, 8].

Short stature remains a major problem among children on KRT. Reduced adult height has been reported in approximately 40% of patients commencing KRT during childhood [9]. Short stature is associated with increased hospitalization [8] and mortality risk [5, 6, 8], mainly due to a higher risk of infectious complications. Interestingly, a study among US pediatric KRT patients [5] reported also a higher mortality risk for tall patients. However, this association was limited to patients with elevated BMI. The mechanisms behind this association are not entirely clear, but taller subjects seem to receive less adequate dialysis [10]. However, as most children are transplanted rapidly this seems to occur far less frequently in children [5].

Both extremes in BMI were associated with higher mortality risk in pediatric KRT [4, 6]. Obesity might also preclude patients from receiving a kidney transplant [4]. Since the increase in obesity in pediatric KRT seems to parallel the global obesity epidemic [11], this might further affect future kidney transplantation rates. Furthermore, obesity might adversely affect (short-term) graft function in children [12–14], but associations are not convergent across all studies [15].

Although associations of anthropometric measures and clinical outcomes might not be causal, poorer outcomes for patients with extreme values for both height and BMI may at least partly reflect nutritional status and severity of illness. Nevertheless, those associations indicate the importance of obtaining a normal body composition for the patients’ daily functioning, not only during childhood, but also later in life.

Reported associations are divergent and mainly based on anthropometric data collected at KRT initiation or from studies in the USA. However, anthropometric measures are easy to obtain during routine clinical visits. Therefore, we aimed to study the association of height and BMI throughout the entire course of childhood KRT with clinical outcomes in a large cohort of European pediatric patients with stage 5 chronic kidney disease (CKD).

## Methods

### Data source and population

Height and weight data were collected within the framework of the ESPN/ERA Registry. On an annual basis the Registry collects individual patient data of all European children requiring KRT [16].

For the present analyses, we included all children < 20 years starting KRT between January 1995 and December 2019 for whom data on height and weight were available, including the following 33 European countries: Albania, Austria, Belarus, Belgium, Bulgaria, Croatia, Czech Republic, Estonia, Finland, France, Georgia, Germany, Hungary, Ireland, Italy, Latvia, Lithuania, Malta, Moldova, Montenegro, the Netherlands, North Macedonia, Norway, Poland, Portugal, Russia, Serbia, Slovakia, Slovenia, Spain, Switzerland, Turkey, and United Kingdom. The Medical Ethics Review Committee of the Amsterdam Medical Center, the Netherlands provided a waiver for ethical approval of this study (W21_257# 21.283).

### Definition of variables

Height was expressed as standard deviation scores (SDS) based on recent national or European growth charts [17]. Short stature was defined as height SDS < –1.88 and tall stature as height SDS > 1.88. BMI was calculated as weight/height^2^ and expressed according to chronological age (0–1.99 year olds) or height-age (≥ 2 year olds) [18]. We defined underweight, overweight and obesity using age- and sex-specific criteria of the World Health Organization (0–1.99 year olds) [19] and the International Obesity Task Force cut-offs (≥ 2 year olds) [20, 21]. Primary renal disease (PRD) and causes of death were categorized according to the ERA coding system. Cardiac failure, cardiac arrest/sudden death, myocardial ischemia and infarction, and cerebrovascular accident were combined as cardiovascular mortality [22].

### Statistical analyses

Access to kidney transplantation, mortality and graft failure risk were calculated as hazard ratios using time-dependent Cox proportional hazards regression models with country as random effect, and adjusting for late entry into the risk set. Whenever appropriate according to the criteria for confounding, sex, PRD, donor type, time-varying age and time-updated KRT modality were included in adjusted models. In the analyses on access to transplantation, only first kidney transplants were considered. Time between KRT start and transplantation was set at 0.5 days for those transplanted pre-emptively. In sensitivity analyses, we tested the effect of reference chart choice by repeating our analyses defining stature and BMI status according to Centers of Disease Control and Prevention (CDC) growth references [23]. As CDC provides reference values for BMI from 2 years onwards, children below 2 years of age were excluded from these analyses. Furthermore, we repeated all analyses expressing BMI according to chronological age rather than height-age for all patients. Since our study has a follow-up of almost 25 years, characteristics and treatment of pediatric KRT patients (e.g. immunosuppression protocols) might have changed during follow-up. Therefore, we also repeated our analyses stratifying by year of KRT initiation: 1995 < 2000 (*N* = 965) and ≥ 2000 (*N* = 10,908). *P*-values < 0.05 were considered statistically significant. All statistical analyses were performed in SAS version 9.4 (SAS Institute Inc., Cary, NC, USA).

## Results

### Patients

Information was available for 11,873 patients, providing 61,906 measurements (median: 4; IQR: 2–7) for a median follow-up of 4.7 (IQR: 2.3–7.9) years. For 62% of patients at least two height and weight measurements were provided. Most patients were male (58.4%), aged between 12 and 19 years when commencing KRT (38.3%), started KRT on peritoneal dialysis (PD) (43.9%) and had congenital anomalies of the kidney and urinary tract (CAKUT) (36.1%) as PRD (Table 1).

**Table 1:** Patient characteristics at initiation of kidney replacement therapy

	Total *N* = 11,873
	No. (%)
*Age (years)*	
0–1.99	1845 (15.5%)
2–5.99	1899 (16.0%)
6–11.99	3587 (30.2%)
12–19.99	4542 (38.3%)
*% Male*	58.4
*First treatment modality*	
HD	4486 (37.8%)
PD	5217 (43.9%)
Kidney transplant	2082 (17.5%)
Unknown dialysis	88 (0.7%)
*Primary renal disease*	
CAKUT	4283 (36.1)
Glomerulonephritis	2095 (17.7)
Cystic kidneys	1421 (12.0)
Hereditary nephropathy	713 (6.0)
Ischemic renal failure	215 (1.8)
HUS	490 (4.1)
Metabolic disease	375 (3.1)
Vasculitis	213 (1.8)
Miscellaneous	1257 (10.6)
Unknown/Missing	811 (6.8)

*HD* hemodialysis; *PD* peritoneal dialysis; *CAKUT* congenital anomalies of the kidney and urinary tract; *HUS* hemolytic uremic syndrome

**Fig. 1:**
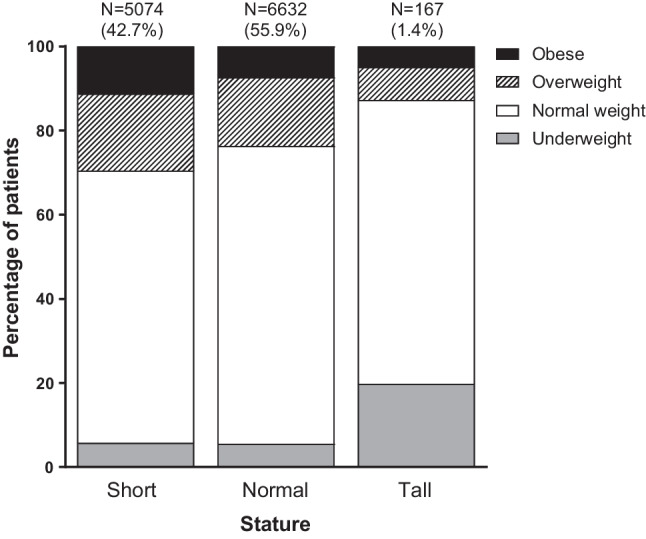
Proportion of patients being underweight, normal weight, overweight and obese stratified by stature

During follow-up, short stature was observed in 42.7% of patients, whereas only 1.4% had a tall stature. Most patients had a normal weight (67.7%), followed by overweight (17.9%), obesity (9.6%) and underweight (4.9%). Overweight and obesity were most common among patients with a short stature, whereas underweight occurred more frequently in tall patients (Fig. 1).

### Access to kidney transplantation

Patients both with short and tall statures had a lower likelihood of kidney transplantation compared with patients with a normal stature, which remained statistically significant after adjustment for age, sex, and PRD (short aHR: 0.82, 95% CI: 0.78–0.86; tall aHR: 0.65, 95% CI: 0.56–0.75) (Table 2).

**Table 2:** Associations between anthropometric measures and access to kidney transplantation

	Unadjusted HR (95% CI)	Adjusted^a^ HR (95% CI)
*Height*		
Short stature	0.86 (0.82–0.90)	0.82 (0.78–0.86)
Normal stature (reference)	1.00	1.00
Tall Stature	0.59 (0.51–0.69)	0.65 (0.56–0.75)
*BMI*		
Underweight	0.75 (0.68–0.83)	0.79 (0.71–0.87)
Normal weight (reference)	1.00	1.00
Overweight	1.14 (1.07–1.22)	1.13 (1.06–1.21)
Obese	0.96 (0.87–1.05)	0.93 (0.84–1.02)

^a^Adjusted for country, sex, age and primary renal disease

Abbreviations: *HR* hazard ratio; *CI* confidence interval; *BMI* body mass index

Compared with normal weight patients, underweight patients (aHR: 0.79, 95% CI: 0.71–0.87) were less likely to receive a kidney transplant. For obese patients we found a similar trend (aHR: 0.93, 95% CI: 0.84–1.02), whereas overweight patients were more likely to receive a kidney transplant compared with normal weight subjects (aHR: 1.13; 95% CI: 1.06–1.21) (Table 2). When stratifying by donor source, we found a lower likelihood of receiving living related donor (LRD) kidneys (aHR: 0.72, 95% CI: 0.59–0.88) and deceased donor (DD) kidneys (aHR: 0.82, 95% CI: 0.73–0.92) for underweight compared with normal weight subjects. Although not statistically significant, a similar trend was seen among obese patients (aHR LRD: 0.89, 95% CI: 0.75–1.06 and aHR DD: 0.95, 95% CI: 0.84–1.07).

Also excluding patients with the lowest chance of receiving a kidney transplant (i.e. patients with a body weight < 10 kg) resulted in a lower likelihood of kidney transplantation for short, tall and underweight patients (Supplemental material).

### Graft failure

Kidney transplant recipients with short (aHR: 1.41, 95% CI: 1.24–1.59) or tall statures (aHR: 1.68, 95% CI: 1.09–2.60) showed an increased risk of graft failure compared with patients having a stature within the normal range. Additional adjustment for body weight or BMI did not attenuate these associations. We did not find any statistically significant associations between BMI categories and graft failure risk, but the graft failure risk for obese children seemed to be of borderline significantly lower risk compared with normal weight children (aHR: 0.81, 95% CI: 0.66–1.01) (Table 3).

**Table 3:** Associations between anthropometric measures and kidney graft failure

	Unadjusted HR (95% CI)	Adjusted^a^ HR (95% CI)
*Height*		
Short stature	1.42 (1.26–1.61)	1.41 (1.24–1.59)
Normal stature (reference)	1.00	1.00
Tall Stature	1.45 (0.93–2.25)	1.68 (1.09–2.60)
*BMI*		
Underweight	1.08 (0.79–1.48)	1.13 (0.83–1.54)
Normal weight (reference)	1.00	1.00
Overweight	0.91 (0.78–1.07)	0.92 (0.78–1.07)
Obese	0.81 (0.66–1.00)	0.81 (0.66–1.01)

^a^Adjusted for country, sex, age, primary renal disease and donor type

Abbreviations: *HR* hazard ratio; *CI* confidence interval; *BMI* body mass index

### Mortality

After a median follow-up of 2.2 years on KRT [IQR:0.9–4.9] 626 patients died (of whom 70% on dialysis). Causes of death were known for 78% of the patients. Most patients died from infections (23.6%) or cardiovascular complications (22.8%).

**Fig. 2:**
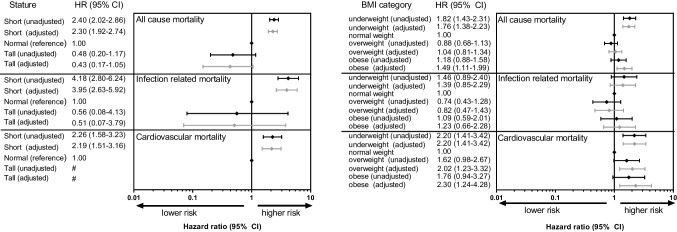
Forest plots for the associations of height (left panel) and BMI (right panel) categories and all cause, infection related, and cardiovascular mortality. The black and grey diamonds and bars represent the unadjusted and adjusted hazard ratios and 95% confidence intervals, respectively. Adjustments were made for country, sex, age, primary renal disease, and treatment modality. # Number of subjects was too low to obtain any effect estimate. Abbreviations: HR, Hazard ratio; CI, confidence interval; BMI, body mass index

Hazard ratios for the associations between height and mortality are presented in Fig. 2 (left panel). Being of short stature was associated with a higher all-cause mortality risk (aHR: 2.30, 95% CI: 1.92–2.74) compared with patients having a stature within the normal range. We did not find such association for patients with tall stature.

Patients with a short stature showed an increased risk of death from infections compared with normal stature patients, independent of country, age, sex, PRD, and treatment modality (aHR: 3.95, 95% CI: 2.63–5.92). The risk of cardiovascular mortality was also higher among patients with a short stature than in those with a normal height (aHR: 2.19, 95% CI: 1.51–3.16). When stratifying by treatment modality most associations remained similar but seemed to be stronger for transplant recipients than for dialysis patients (Supplemental table).

All-cause mortality risk was higher among underweight (aHR: 1.76, 95% CI: 1.38–2.23) than among normal weight patients (Fig. 2, right panel). Obesity, but not overweight, was associated with a higher all-cause mortality risk (aHR: 1.49, 95% CI: 1.11–1.99). Compared with normal weight patients, underweight (aHR: 2.20, 95% CI: 1.41–3.42), overweight (aHR: 2.02, 95% CI: 1.23–3.32) and obese (aHR: 2.30, 95% CI: 1.24–4.28) patients had an increased risk of death from cardiovascular disease (Fig. 2, right panel).

### Sensitivity analyses

The results of our sensitivity analyses are provided in the Supplemental material. Neither the association between stature and access to transplantation, nor the association between stature and mortality was affected by growth reference chart choice, as similar results were obtained when height status was categorized according to CDC growth references. Similarly, we did not find any different associations for BMI based on CDC reference charts. Moreover, BMI for chronological age rather than BMI for height-age yielded similar results as the main analyses. Analyses stratified by year of KRT initiation (before or after the year 2000) also yielded similar results as our main analyses (Supplementary tables).

## Discussion

Our pan-European study in pediatric KRT patients, suggests that both height and BMI have substantial impact on kidney transplantation, graft failure and mortality risk. We found a higher mortality risk among patients with a short stature or those who were underweight throughout the course of pediatric KRT. Extremes in both height and BMI were associated with a lower likelihood of receiving a kidney transplant and graft failure was higher among short and tall patients.

Abnormal height and body composition are common complications of childhood CKD [24], simultaneously they are risk factors for poor outcomes after (pediatric) KRT, like increased hospitalizations [8], increased mortality [4–6, 8], and lower quality of life [7]. Given the impact of anthropometric measures on patient outcomes after pediatric KRT, these measures can assist in finding the most vulnerable patients and tailor treatment accordingly.

### Access to kidney transplantation

Irrespective of reference chart, we consistently found lower kidney transplantation rates for short and underweight patients compared with patients of a normal stature or weight, respectively. Also, after excluding patients with a body weight below 10 kg, generally considered unsuitable transplant candidates because of technical reasons [25], kidney transplantation rates were lower in short and underweight patients. This is not surprising, as underweight and short patients are likely to be the sickest patients.

Obese patients also had a lower likelihood of kidney transplantation. Obesity can be a contra-indication for transplantation in adult nephrology care because of increased risk for complications and worse transplant outcomes [26], but seems uncommon in pediatric nephrology [27]. However, as obesity runs within families, difficulty in finding suitable LRD kidneys for obese patients [28] might contribute to this finding. Stratifying for donor source did not show any differences in access to kidneys from deceased or living donors among obese patients, suggesting that other factors are involved, such as overall health status of donors and recipients. Unfortunately, we were not able to investigate this in more detail.

Surprisingly, overweight patients were more likely to receive a kidney transplant than normal weight patients. We postulate that these patients were probably considered ‘well-nourished’ and not so much high-risk patients. Another explanation might be country differences in both prevalence of overweight and access to kidney transplantation among dialysis patients. Previous registry studies showed that dialysis patients from Finland, Spain and United Kingdom were more likely to be overweight [18] and those same countries all have a very good access to kidney transplantation [29]. Although we adjusted our analyses for country, there might be some residual confounding.

### Graft failure

Kidney graft failure was higher among short and tall patients. A recent CKiD study reported that patients with short statures prior to kidney transplantation had a 40% faster progression to eGFR < 45 ml/min/1.73 m^2^ post-transplant [13]. The authors speculated that mineral metabolism, chronic inflammation, and poor nutrition might contribute to poor transplant outcomes in children with growth failure [13]. On the other hand, it is unclear why tall children had a higher graft failure risk than children of normal height. This association was independent of body weight or BMI. Tall stature might be the result of overgrowth syndromes associated with poor outcomes. While for some of the tall patients information on extra-renal comorbidities was listed, including disorders of sex development, neurofibromatosis I and Marfan syndrome, the overall information on comorbidities included in the registry was too limited to study in more detail. Although we adjusted our analyses for age, the proportion of children younger than 2 years commencing KRT was much higher among tall subjects (34%) than among short or normal height patients (both 7%), an age group with poorer kidney graft survival [30]. Given the low number of tall subjects included (*N* = 167) this warrants further exploration.

Underweight, overweight or obesity were not risk factors for graft failure. Other pediatric studies reported conflicting results. While Kaur et al. reported higher risk for graft failure among overweight and obese patients, being underweight seemed to be protective [14]. An ANZDATA Registry study found a higher graft failure risk among obese, but not among underweight or overweight patients [15]. Both studies based their conclusions on pre-transplant BMI only, whereas we included all available BMI measurements to estimate its effect on kidney graft failure. Moreover, a meta-analysis among adult patients with stage 5 CKD reported that obesity was a risk factor for graft loss before the year 2000, but after 2000 graft survival of obese and non-obese patients was similar [31]. In our cohort most patients were transplanted after 2000, and our sensitivity analyses stratifying by year of KRT start before or after the year 2000 yielded similar results compared with the full cohort.

### Stature and mortality

Short patients showed an increased mortality risk, independent of country, age, sex, and treatment modality. Both mortality due to infections and cardiovascular mortality were significantly higher among patients with a short stature. These findings are in line with previous studies on height at KRT initiation [5, 6, 8]. It is unlikely that poor growth itself causes increased mortality, but as a marker of disease severity or nutritional status, it has been associated with reduced health-related quality of life [32] and might predispose to infections [8]. Moreover, growth failure could also be caused by genetic factors, including syndromic disorders associated with CKD [24] and as such being associated with increased mortality.

Tall stature was not associated with mortality. In contrast to our findings, US studies in both pediatric and adult KRT patients reported an increased risk of death for patients with a tall stature at KRT initiation [5, 10, 33]. However, among US pediatric patients the increased mortality in tall subjects was limited to white and/or obese patients [5]. The authors suggested that this association might be due to higher malignancy-related death, possibly due to higher lifetime exposure to immunosuppressive drugs. In our study none of the patients who died of malignancies had a tall stature.

### BMI and mortality

Being a marker of malnutrition and/or severity of illness [4, 6], it was not surprising to find an increased all-cause mortality risk for underweight patients. Like US studies [4, 6], we found an association between obesity and all-cause mortality risk. However, when Ku et al. [4] additionally adjusted their models for kidney transplantation as a time-dependent variable, the higher mortality risk among obese patients was attenuated, and our models were adjusted for time-varying treatment modality. This difference can be partly explained by the timing of BMI measurements. Ku et al. only included BMI measurements at first KRT, whereas our analyses included all available BMI measurements throughout childhood KRT. Moreover, the authors expressed BMI according to chronological age, but our sensitivity analyses with BMI for chronological age yielded similar results as our main analyses on BMI for height-age. Different case mix of patient populations in Europe and North America could also have contributed to these differences.

Interestingly, we also found an increased risk of cardiovascular mortality among obese patients, suggesting that BMI during childhood KRT already has cardiovascular impact. Indeed, in studies among pediatric CKD patients and children treated with PD, adiposity was associated with several markers of cardiovascular disease, such as left ventricular hypertrophy [34] and arterial stiffness [35]. Although it seems to be beneficial for cardiovascular health to lose weight, a recent study among US children treated with KRT found an increased mortality risk in patients with a large annual BMI decrease, but not for those whose BMI decreased moderately [36].

### Strengths and limitations

Strengths of this study include the large sample size including height and weight measurements during the entire course of childhood KRT and the multinational data acquisition across Europe. This enabled us to assess associations of anthropometric markers and hard clinical outcomes. Furthermore, obtaining similar results through several sensitivity analyses, including the comparison between previous and current eras of pediatric kidney transplantation, strengthens our conclusion that despite improved quality of care sicker patients (e.g. short stature and underweight patients) universally show poorer outcomes after pediatric KRT. Nevertheless, some limitations inherent to the study design need to be acknowledged. Due to the observational nature of the ESPN/ERA Registry, we cannot prove causation. Moreover, BMI as a marker of adiposity does not differentiate between lean and fat body mass. However, it is the most used marker of obesity in childhood, and a recent study among pediatric CKD patients in the US found that BMI and waist circumference showed good agreement in associations with several metabolic and cardiovascular markers [37]. Another limitation of our study was the lack of detailed data on growth hormone treatment and immunosuppressive regimens including corticosteroid therapy, as well as limited information on extra-renal comorbidities.

In summary, we found that short and tall stature and both extremes in BMI among pediatric KRT patients were associated with decreased likelihood of kidney transplantation and a greater mortality risk, especially from cardiovascular causes. Obesity might thus have substantial impact on cardiovascular risk among children treated with KRT. As cardiovascular risk factors track from childhood to adulthood, pediatric obesity might have an even stronger cardiovascular impact when follow-up was extended into adulthood. Therefore, our findings add to the existing evidence that lifestyle modification, nutritional management, and growth hormone treatment in persistent short stature is of paramount importance in these patients.

## Supplementary Information

Below is the link to the electronic supplementary material.

Graphical Abstract (PPTX 60 KB)

Supplementary file2 (DOCX 44 KB)

## Data Availability

The data underlying this manuscript cannot be shared with any third party because the national registries that provided data to the ESPN/ERA Registry remain the owners of the data.
