# Supplementary material for: Associations of longitudinal height and weight with clinical outcomes in pediatric kidney replacement therapy: results from the ESPN/ERA Registry
Source: Pediatr Nephrol. 2023 May 8;38(10):3435–43. doi: 10.1007/s00467-023-05973-3 (PMC10465625; doi:10.1007/s00467-023-05973-3)
Supplement: Supplementary file 1 — Graphical Abstract (PPTX 60 KB) [file 467_2023_5973_MOESM1_ESM.pptx]

## Slide 1
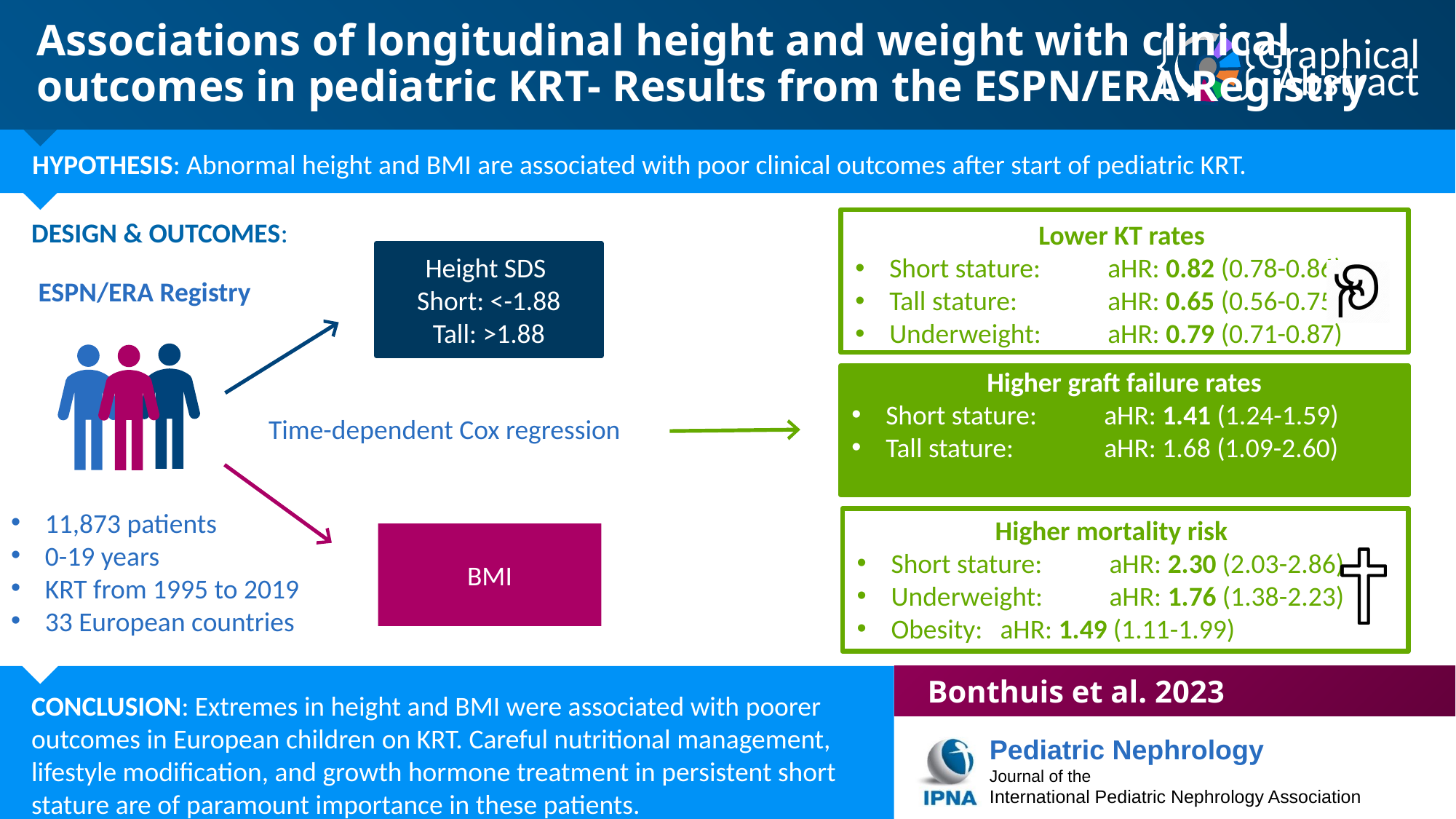

Associations of longitudinal height and weight with clinical
outcomes in pediatric KRT- Results from the ESPN/ERA Registry
HYPOTHESIS: Abnormal height and BMI are associated with poor clinical outcomes after start of pediatric KRT.
DESIGN & OUTCOMES:
Lower KT rates
Short stature: 	aHR: 0.82 (0.78-0.86)
Tall stature: 	aHR: 0.65 (0.56-0.75)
Underweight:	aHR: 0.79 (0.71-0.87)
Height SDS
Short: <-1.88
Tall: >1.88
ESPN/ERA Registry
Higher graft failure rates
Short stature:	aHR: 1.41 (1.24-1.59)
Tall stature:	aHR: 1.68 (1.09-2.60)
Time-dependent Cox regression
11,873 patients
0-19 years
KRT from 1995 to 2019
33 European countries
Higher mortality risk
Short stature: 	aHR: 2.30 (2.03-2.86)
Underweight:	aHR: 1.76 (1.38-2.23)
Obesity:	aHR: 1.49 (1.11-1.99)
BMI
Bonthuis et al. 2023
CONCLUSION: Extremes in height and BMI were associated with poorer outcomes in European children on KRT. Careful nutritional management, lifestyle modification, and growth hormone treatment in persistent short stature are of paramount importance in these patients.
