# Supplementary material for: Associations of longitudinal height and weight with clinical outcomes in pediatric kidney replacement therapy: results from the ESPN/ERA Registry
Source: Pediatr Nephrol. 2023 May 8;38(10):3435–43. doi: 10.1007/s00467-023-05973-3 (PMC10465625; doi:10.1007/s00467-023-05973-3)
Supplement: Supplementary file 2 — Supplementary file2 (DOCX 44 KB) [file 467_2023_5973_MOESM2_ESM.docx]

**Supplementary tables** (Sensitivity analyses)

**Supplementary Table 1. Associations of height and BMI with access to kidney transplantation excluding patients with a body weight below 10 kg**

|  | **Unadjusted**  **HR (95% CI)** | **Adjusted^a^**  **HR (95% CI)** |
| --- | --- | --- |
| **Height** |  |  |
| Short stature | 0.87 (0.83–0.92) | 0.83 (0.79–0.87) |
| Normal stature (reference) | 1.00 | 1.00 |
| Tall Stature | 0.52 (0.44–0.61) | 0.59 (0.50–0.69) |
| **BMI** |  |  |
| Underweight | 0.77 (0.69–0.86) | 0.81 (0.72–0.90) |
| Normal weight (reference) | 1.00 | 1.00 |
| Overweight | 1.13 (1.05–1.20) | 1.12 (1.05–1.20) |
| Obese | 0.93 (0.84–1.02) | 0.91 (0.83–1.00) |

^a^Adjusted for country, sex, age, and primary renal disease

Abbreviations: BMI, body mass index; kg, kilogram; HR, hazard ratio; CI, confidence interval**Supplementary Table 2. Association of height and mortality stratified by treatment modality**

|  | **Dialysis patients** | | **Kidney transplant recipients** | |
| --- | --- | --- | --- | --- |
|  | **Unadjusted**  **HR (95% CI)** | **Adjusted^1^**  **HR (95% CI)** | **Unadjusted**  **HR (95% CI)** | **Adjusted^a^**  **HR (95% CI)** |
| **All-cause mortality** |  |  |  |  |
| Short stature | 1.87 (1.55–2.25) | 1.98 (1.64–2.39) | 2.76 (1.98–3.86) | 2.80 (2.00–3.93) |
| Normal stature (reference) | 1.00 | 1.00 | 1.00 | 1.00 |
| Tall stature | 0.46 (0.20–1.05) | 0.49 (0.21–1.11) | 1.28 (0.18–9.35) | 1.19 (0.16–8.78) |
| **Infection related mortality** |  |  |  |  |
| Short stature | 2.76 (1.83–4.16) | 2.80 (1.85–4.24) | 4.37 (2.26–8.46) | 4.29 (2.21–8.33) |
| Normal stature (reference) | 1.00 | 1.00 | 1.00 | 1.00 |
| Tall stature | 0.35 (0.05–2.66) | 0.38 (0.05–2.83) | # | # |
| **Cardiovascular mortality** |  |  |  |  |
| Short stature | 1.98 (1.35–2.90) | 2.17 (1.47–3.21) | 2.05 (0.87–4.86) | 2.15 (0.91–5.08) |
| Normal stature (reference) | 1.00 | 1.00 | 1.00 | 1.00 |
| Tall stature | 0.34 (0.05–2.55) | 0.38 (0.05–2.81) | # | # |

^a^Adjusted for country, sex, age, and primary renal disease.

#Number of subjects was too small to obtain an effect estimate.

Abbreviations: HR, hazard ratio; CI, confidence interval.**Supplementary Table 3. Anthropometry and access to kidney transplantation**

|  | **IOTF cut-offs** | | **CDC growth charts^a^** | |
| --- | --- | --- | --- | --- |
|  | **Unadjusted**  **HR (95% CI)** | **Adjusted^b^**  **HR (95% CI)** | **Unadjusted**  **HR (95% CI)** | **Adjusted^b^**  **HR (95% CI)** |
| **Height** |  | |  | |
| Short stature |  |  | 0.84 (0.80–0.88) | 0.80 (0.76–0.84) |
| Normal stature (reference) |  |  | 1.00 | 1.00 |
| Tall Stature |  |  | 0.57 (0.49–0.65) | 0.62 (0.54–0.72) |
| **BMI (height-age)** |  | |  | |
| Underweight |  |  | 0.79 (0.74–0.85) | 0.83 (0.77–0.88) |
| Normal weight (reference) |  |  | 1.00 | 1.00 |
| Overweight |  |  | 1.06 (0.98–1.14) | 1.04 (0.97–1.13) |
| Obese |  |  | 0.96 (0.88–1.04) | 0.95 (0.87–1.03) |
| **BMI (chronological age)** |  | |  | |
| Underweight | 0.75 (0.69–0.82) | 0.77 (0.70–0.84) | 0.84 (0.79–0.90) | 0.83 (0.77–0.89) |
| Normal weight (reference) | 1.00 | 1.00 | 1.00 | 1.00 |
| Overweight | 1.04 (0.97–1.12) | 1.05 (0.98–1.13) | 1.06 (0.98–1.14) | 1.03 (0.95–1.12) |
| Obese | 0.93 (0.83–1.04) | 0.90 (0.80–1.00) | 1.01 (0.92–1.10) | 0.96 (0.88–1.05) |

*^a^*CDC growth charts were used excluding patients below 2 years; ^b^Adjusted for country, sex, age, and primary renal disease.

Abbreviations: IOTF, International Obesity Taskforce; CDC, Centers for Disease Control and Prevention; HR, hazard ration; CI, confidence interval; BMI, body mass index.

**Supplementary Table 4. Associations between stature according to CDC growth charts and mortality**

|  | **CDC growth charts** | |
| --- | --- | --- |
|  | **Unadjusted**  **HR (95% CI)** | **Adjusted^a^**  **HR (95% CI)** |
| **All-cause mortality** |  |  |
| Short stature | 2.41 (2.05–2.84) | 2.29 (1.94–2.70) |
| Normal stature (reference) | 1.00 | 1.00 |
| Tall Stature | 0.39 (0.15–1.06) | 0.35 (0.13–0.95) |
| **Infection related mortality** |  |  |
| Short stature | 3.25 (2.30–4.61) | 3.04 (2.13–4.33) |
| Normal stature (reference) | 1.00 | 1.00 |
| Tall stature | 0.45 (0.06–3.31) | 0.42 (0.06–3.08) |
| **Cardiovascular mortality** |  |  |
| Short stature | 2.42 (1.72–3.41) | 2.37 (1.66–3.37) |
| Normal stature (reference) | 1.00 | 1.00 |
| Tall stature | # | # |

^a^Adjusted for country, sex, age, primary renal disease, and treatment modality.

# Number of subjects was too small to obtain an effect estimate.

Abbreviations: CDC, Centers for Disease Control and Prevention; HR, hazard ratio; CI, confidence interval.

**Supplementary Table** **5. Associations between BMI and mortality according to different cut-offs**

|  | **IOTF cut-offs** | | **CDC growth charts^a^** | |
| --- | --- | --- | --- | --- |
|  | **Unadjusted**  **HR (95% CI)** | **Adjusted^b^**  **HR (95% CI)** | **Unadjusted**  **HR (95% CI)** | **Adjusted^b^**  **HR (95% CI)** |
| ***Height-age*** |  |  |  |  |
| **All-cause mortality** |  |  |  |  |
| Underweight |  |  | 1.98 (1.57–2.51) | 1.79 (1.42–2.26) |
| Normal weight (reference) |  |  | 1.00 | 1.00 |
| Overweight |  |  | 0.72 (0.52–1.00) | 0.87 (0.63–1.22) |
| Obese |  |  | 1.25 (0.96–1.64) | 1.58 (1.21–2.07) |
| **Infection related mortality** |  |  |  |  |
| Underweight |  |  | 1.55 (0.96–2.53) | 1.45 (0.88–2.37) |
| Normal weight (reference) |  |  | 1.00 | 1.00 |
| Overweight |  |  | 0.62 (0.31–1.25) | 0.68 (0.34–1.37) |
| Obese |  |  | 1.09 (0.62–1.92) | 1.22 (0.69–2.17) |
| **Cardiovascular mortality** |  |  |  |  |
| Underweight |  |  | 2.39 (1.45–3.93) | 2.13 (1.29–3.52) |
| Normal weight (reference) |  |  | 1.00 | 1.00 |
| Overweight |  |  | 1.10 (0.55­–2.22) | 1.51 (0.75–3.02) |
| Obese |  |  | 2.05 (1.16–3.62) | 2.87 (1.62–5.11) |
|  |  |  |  |  |
| ***Chronological age*** |  |  |  |  |
| **All-cause mortality** |  |  |  |  |
| Underweight | 2.13 (1.72–2.65) | 1.96 (1.58–2.43) | 2.37 (1.91–2.95) | 2.01 (1.61–2.51) |
| Normal weight (reference) | 1.00 | 1.00 | 1.00 | 1.00 |
| Overweight | 0.85 (0.64–1.12) | 0.98 (0.74–1.30) | 0.81 (0.59–1.12) | 0.96 (0.69–1.32) |
| Obese | 1.17 (0.83–1.66) | 1.44 (1.02–2.05) | 1.14 (0.84–1.55) | 1.39 (1.02–1.90) |
| **Infection related mortality** |  |  |  |  |
| Underweight | 1.66 (1.05–2.63) | 1.53 (0.97–2.43) | 2.59 (1.68–4.02) | 2.28 (1.46–3.57) |
| Normal weight (reference) | 1.00 | 1.00 | 1.00 | 1.00 |
| Overweight | 0.72 (0.39–1.31) | 0.79 (0.43–1.46) | 0.98 (0.53–1.82) | 1.08 (0.58–2.02) |
| Obese | 1.11 (0.54–2.30) | 1.24 (0.60–2.58) | 0.96 (0.47–1.92) | 1.08 (0.53–2.19) |
| **Cardiovascular mortality** |  |  |  |  |
| Underweight | 2.32 (1.52–3.54) | 2.19 (1.44–3.34) | 2.16 (1.36–3.44) | 1.77 (1.09–2.86) |
| Normal weight (reference) | 1.00 | 1.00 | 1.00 | 1.00 |
| Overweight | 1.55 (0.91–2.63) | 1.85 (1.09–3.15) | 0.97 (0.48–1.96) | 1.25 (0.61–2.53) |
| Obese | 1.62 (0.78–3.36) | 2.02 (0.97–4.23) | 1.74 (0.96–3.17) | 2.21 (1.20–4.10) |

^a^For CDC growth charts patients below 2 years of age were excluded. ^b^Adjusted for country, sex, age, primary renal disease, and treatment modality.

Abbreviations: BMI, body mass index; IOTF, International Obesity Taskforce; CDC, Centers for Disease Control and Prevention; HR, hazard ratio; CI, confidence interval.

**Supplementary Table 6. Associations between anthropometric measures and access to deceased donor transplantation**

|  | **Unadjusted**  **HR (95% CI)** | **Adjusted^a^**  **HR (95% CI)** |
| --- | --- | --- |
| **Height** |  |  |
| Short stature | 0.91 (0.85–0.97) | 0.86 (0.80–0.92) |
| Normal stature (reference) | 1.00 | 1.00 |
| Tall Stature | 0.67 (0.56–0.82) | 0.77 (0.62–0.92) |
| **BMI** |  |  |
| Underweight | 0.79 (0.70–0.91) | 0.83 (0.73–0.95) |
| Normal weight (reference) | 1.00 | 1.00 |
| Overweight | 1.14 (1.03–1.26) | 1.12 (1.01–1.24) |
| Obese | 0.91 (0.77–1.06) | 0.88 (0.76–1.03) |

^a^Adjusted for country, sex, age, and primary renal disease.

Abbreviations: HR, hazard ratio; CI, confidence interval; BMI, body mass index.

**Supplementary Table 7. Associations between anthropometric measures and access to living donor transplantation**

|  | **Unadjusted**  **HR (95% CI)** | **Adjusted^a^**  **HR (95% CI)** |
| --- | --- | --- |
| **Height** |  |  |
| Short stature | 0.71 (0.63–0.80) | 0.67 (0.41–0.75) |
| Normal stature (reference) | 1.00 | 1.00 |
| Tall Stature | 0.32 (0.22–0.47) | 0.41 (0.28–0.59) |
| **BMI** |  |  |
| Underweight | 0.73 (0.57–0.93) | 0.79 (0.61–0.99) |
| Normal weight (reference) | 1.00 | 1.00 |
| Overweight | 1.04 (0.88–1.23) | 1.00 (0.85–1.18) |
| Obese | 0.85 (0.65–1.10) | 0.80 (0.62–1.04) |

^a^Adjusted for country, sex, age, and primary renal disease;

Abbreviations: HR, hazard ratio; CI, confidence interval; BMI, body mass index.

**Supplementary Table 8.** **Associations between anthropometric measures and access to kidney transplantation stratified by period of KRT start**

|  | **KRT start 1995 < 2000** | | **KRT start 2000–2019** | |
| --- | --- | --- | --- | --- |
|  | Unadjusted  HR (95% CI) | **Adjusted^a^**  **HR (95% CI)** | **Unadjusted**  **HR (95% CI)** | **Adjusted^a^**  **HR (95% CI)** |
| **Height** |  |  |  |  |
| Short stature | 0.97 (0.83–1.13) | 0.83 (0.71–0.97) | 0.85 (0.81–0.90) | 0.81 (0.77–0.85) |
| Normal stature (reference) | 1.00 | 1.00 | 1.00 | 1.00 |
| Tall Stature | 1.57 (0.86–2.88) | 1.41 (0.76–2.63) | 0.55 (0.47–0.64) | 0.62 (0.53–0.72) |
| **BMI** |  |  |  |  |
| Underweight | 0.73 (0.51–1.05) | 0.70 (0.48–1.00) | 0.75 (0.68–0.83) | 0.80 (0.72–0.88) |
| Normal weight (reference) | 1.00 | 1.00 | 1.00 | 1.00 |
| Overweight | 0.82 (0.65–1.04) | 0.91 (0.72–1.15) | 1.18 (1.10–1.27) | 1.16 (1.08–1.24) |
| Obese | 0.83 (0.62–1.11) | 0.80 (0.59–1.07) | 0.98 (0.89–1.09) | 0.95 (0.85–1.05) |

^a^Adjusted for country, sex, age and primary renal disease.

Abbreviations: KRT, kidney replacement therapy; HR, hazard ratio; CI, confidence interval; BMI, body mass index.

**Supplementary Table 9.** **Associations between anthropometric measures and kidney graft failure stratified by period of KRT start**

|  | | **KRT start 1995 < 2000** | | **KRT start 2000–2019** | |
| --- | --- | --- | --- | --- | --- |
|  | **Unadjusted**  **HR (95% CI)** | | **Adjusted^a^**  **HR (95% CI)** | **Unadjusted**  **HR (95% CI)** | **Adjusted^a^**  **HR (95% CI)** |
| **Height** |  | |  |  |  |
| Short stature | 1.45 (1.07-1.96) | | 1.48 (1.09-2.01) | 1.42 (1.24-1.62) | 1.38 (1.21-1.59) |
| Normal stature (reference) | 1.00 | | 1.00 | 1.00 | 1.00 |
| Tall Stature | # | | # | 1.39 (0.84-2.19) | 1.52 (0.94-2.47) |
| **BMI** |  | |  |  |  |
| Underweight | 0.62 (0.19-2.02) | | 0.61 (0.18-2.04) | 1.17 (0.84-1.61) | 1.22 (0.88-1.68) |
| Normal weight (reference) | 1.00 | | 1.00 | 1.00 | 1.00 |
| Overweight | 0.50 (0.33-0.76) | | 0.49 (0.32-0.75) | 1.04 (0.87-1.23) | 1.04 (0.88-1.24) |
| Obese | 0.42 (0.25-0.72) | | 0.42 (0.25-0.72) | 0.94 (0.75-1.18) | 0.94 (0.75-1.19) |

^a^Adjusted for country, sex, age, primary renal disease and donor type.

#Number of subjects was too small to obtain an effect estimate.

Abbreviations: KRT, kidney replacement therapy; HR, hazard ratio; CI, confidence interval; BMI, body mass index.

**Supplementary Table 10. Association of height and mortality stratified by period of KRT start**

|  | **KRT start 1995 < 2000** | | **KRT start 2000–2019** | |
| --- | --- | --- | --- | --- |
|  | **Unadjusted**  **HR (95% CI)** | **Adjusted^a^**  **HR (95% CI)** | **Unadjusted**  **HR (95% CI)** | **Adjusted^a^**  **HR (95% CI)** |
| **All-cause mortality** |  |  |  |  |
| Short stature | 1.87 (1.12–3.13) | 1.96 (1.16–3.34) | 2.50 (2.08–3.00) | 2.35 (1.95–2.84) |
| Normal stature (reference) | 1.00 | 1.00 | 1.00 | 1.00 |
| Tall stature | # | # | 0.50 (0.20–1.23) | 0.45 (0.18–1.09) |
| **Infection related mortality** |  |  |  |  |
| Short stature | # | # | 4.43 (2.89–6.80) | 4.15 (2.69–6.41) |
| Normal stature (reference) | # | # | 1.00 | 1.00 |
| Tall stature | # | # | 0.61 (0.08–4.56) | 0.55 (0.07–4.14) |
| **Cardiovascular mortality** |  |  |  |  |
| Short stature | # | # | 2.33 (1.60–3.40) | 2.24 (1.52–3.30) |
| Normal stature (reference) | # | # | 1.00 | 1.00 |
| Tall stature | # | # | # | # |

^a^Adjusted for country, sex, age, primary renal disease, and treatment modality.

#Number of subjects was too small to obtain an effect estimate.

Abbreviations: KRT, kidney replacement therapy; HR, hazard ratio; CI, confidence interval

**Supplementary Table 11. Association of BMI and mortality stratified by period of KRT start**

|  | **KRT start 1995 < 2000** | | **KRT start 2000–2019** | |
| --- | --- | --- | --- | --- |
|  | **Unadjusted**  **HR (95% CI)** | **Adjusted^a^**  **HR (95% CI)** | **Unadjusted**  **HR (95% CI)** | **Adjusted^a^**  **HR (95% CI)** |
| **All-cause mortality** |  |  |  |  |
| Underweight | 2.23 (0.92–5.47) | 2.54 (1.03–6.22) | 1.78 (1.39–2.29) | 1.70 (1.33–2.17) |
| Normal weight (reference) | 1.00 | 1.00 | 1.00 | 1.00 |
| Overweight | 0.97 (0.48–1.98) | 1.05 (0.51–2.18) | 0.89 (0.68–1.17) | 1.06 (0.80–1.39) |
| Obese | 1.49 (0.73–3.06) | 1.65 (0.79–3.48) | 1.14 (0.83–1.57) | 1.45 (1.05–2.01) |
| **Infection related mortality** |  |  |  |  |
| Underweight | # | # | 1.40 (0.83–2.35) | 1.32 (0.78–2.22) |
| Normal weight (reference) | # | # | 1.00 | 1.00 |
| Overweight | # | # | 0.68 (0.37–1.26) | 0.75 (0.40–1.38) |
| Obese | # | # | 1.07 (0.55–2.08) | 1.19 (0.61–2.33) |
| **Cardiovascular mortality** |  |  |  |  |
| Underweight | # | # | 2.04 (1.27–3.27) | 2.01 (1.26–3.22) |
| Normal weight (reference) | # | # | 1.00 | 1.00 |
| Overweight | # | # | 1.85 (1.11–3.08) | 2.23 (1.34–3.71) |
| Obese | # | # | 1.83 (0.96–3.50) | 2.39 (1.25–4.56) |

^a^Adjusted for country, sex, age, primary renal disease, and treatment modality.

#Number of subjects was too small to obtain an effect estimate.

Abbreviations: KRT, kidney replacement therapy; HR, hazard ratio; CI, confidence interval; BMI, body mass index
